# Elevated ATP via enhanced miRNA-30b, 30c, and 30e downregulates the expression of CD73 in CD8^+^ T cells of HIV-infected individuals

**DOI:** 10.1371/journal.ppat.1010378

**Published:** 2022-03-24

**Authors:** Shima Shahbaz, Isobel Okoye, Gregg Blevins, Shokrollah Elahi

**Affiliations:** 1 School of Dentistry, Division of Foundational Sciences, Faculty of Medicine and Dentistry, University of Alberta, Edmonton, Canada; 2 Department of Medicine, Division of Neurology, Faculty of Medicine and Dentistry, University of Alberta, Edmonton, Canada; 3 Department of Oncology, Faculty of Medicine and Dentistry, University of Alberta, Edmonton, Canada; 4 Li Ka Shing Institute of Virology, Faculty of Medicine and Dentistry, University of Alberta, Edmonton, Canada; Vaccine Research Center, UNITED STATES

## Abstract

CD8^+^ T cells play a crucial role against chronic viral infections, however, their effector functions are influenced by the expression of co-stimulatory/inhibitory receptors. For example, CD73 works with CD39 to convert highly inflammatory ATP to adenosine. However, its expression on T cells in the context of viral infections has not been well defined. Here, we analyzed the expression of CD73 on human T cells in a cohort of 102 HIV-infected individuals including those on antiretroviral therapy (ART), ART-naïve, and long-term non-progressors who were not on ART. We found that the frequency of CD73^+^ T cells was markedly lower among T cell subsets (e.g. naïve, effector or memory) in the peripheral blood of all HIV-infected individuals. Notably, CD73 was decreased at the cell surface, intracellular and gene levels. Functionally, CD8^+^CD73^+^ T cells exhibited decreased cytokine expression (TNF-α, IFN-γ and IL-2) upon global or antigen-specific stimulation and impaired expression of cytolytic molecules at the gene and protein levels. In contrast, CD8^+^CD73^+^ T cells expressed elevated levels of homing receptors such as CCR7, α4β7 integrin, which suggests a migratory advantage for these cells as observed *in vitro*. We also observed significant migration of CD73^+^CD8^+^ T cells into the cerebrospinal fluids of multiple sclerosis (MS) patients at the time of disease relapse. Moreover, we found that elevated levels of ATP in the plasma of HIV-infected individuals upregulates the expression of miRNA30b-e in T cells *in vitro*. In turn, inhibition of miRNAs (30b, 30c and 30e) resulted in significant upregulation of CD73 mRNA in CD8^+^ T cells. Therefore, we provide a novel mechanism for the downregulation of CD73 via ATP-induced upregulation of miRNA30b, 30c and 30e in HIV infection. Finally, these observations imply that ATP-mediated downregulation of CD73 mainly occurs via its receptor, P2X1/P2RX1. Our results may in part explain why HIV-infected individuals have reduced risk of developing MS considering the role of CD73 for efficient T cell entry into the central nervous system.

## Author summary

CD8^+^ T cells (killer T cells) play an important role against chronic viral infections, however, their functional properties get compromised during the course of HIV infection. CD73, is one of molecules that influences T cell functions, however, its role in the context of viral infections has not been well defined. Here, we analyzed the expression of CD73 on T cells in a cohort of 102 HIV-infected individuals including those on antiretroviral therapy (ART), ART-naïve, and long-term non-progressors who were not on ART. We found that the frequency of T cells expressing this molecule was markedly lower among different T cell subsets obtained from the blood of HIV-infected individuals. Notably, CD73 was decreased at the intracellular protein and gene levels. Furthermore, we found that T cells expressing this molecule (CD73) had impaired functional properties. In contrast, we observed that T cells expressing CD73 had elevated levels of homing receptors, which suggests a migratory advantage for these cells. This was also supported by increased CD73^+^ T cells in the cerebrospinal fluids of multiple sclerosis patients when they experienced disease replace. Moreover, we found that the elevated level of ATP in the plasma of HIV-infected individuals is responsible for the upregulation of miRNA30b, 30c and 30e, resulting in reduced expression of CD73.

## Introduction

With the success of antiretroviral therapy (ART), the prognosis of HIV infection has substantially improved [1]. As such HIV infection is no longer considered a fatal disease but instead a chronic and manageable infection [2]. Human HIV infection is suppressed but not eliminated by ART, as a result, viral antigen persistence is a major cause of substantial immune activation and chronic inflammation, both of which are associated with ‘inflammaging’[3]. This chronic immune activation is linked to microbial translocation and increased levels of coagulation factors contributing to non-HIV-related comorbidities in HIV-infected individuals [4]. As such, recent research has focused on identifying the mechanism(s) underlying immune activation and how it can be prevented.

CD73 (NT5E), an ectoenzyme, is expressed on the surface of various immune cells, including monocytes, dendritic cells, neutrophils, B and T cells [5]. CD73 works mainly with CD39, another cell surface ectoenzyme to convert highly inflammatory ATP to immunosuppressive adenosine in a tightly regulated manner [6]. While the conversion of ATP to ADP and subsequently AMP is catalyzed by CD39, CD73 catalyzes the conversion of AMP into adenosine [6]. In addition to its enzymatic activity, CD73 functions as a co-inhibitory molecule on T cells, reducing the threshold for naïve T cells’ activation following antigen encounter [7]. Also, CD73 acts as an adhesion molecule on T cells, mediating lymphocyte binding to endothelial cells through engagement with the Lymphocyte Function-Associated Antigen-1 (LFA-1) [8]. The CD73 gene is located on the long arm of chromosome 6 and its expression is controlled by several transcriptional factors [9]. The promotor region of CD73 has binding sites for transcriptional factors SP1, AP-2, cAMP-responsive elements, as well as SMAD proteins [10,11]. Besides, the hypoxia-inducible factor (HIF-1) can elevate CD73 expression by direct binding to the CD73 promoter, which accounts for the functional role of CD73 in hypoxia adaptation [12,13]. Moreover, different cytokines may impact CD73 gene expression directly or through the regulation of other transcriptional factors. For example, while IL-6 enhances CD73 expression through STAT3 activation, TGF-β represses CD73 expression via the suppression of transcriptional factor Gfi-1 [14]. Finally, FOXP3 the transcriptional factor of regulatory T cells (Tregs) has been shown to enhance CD73 expression in murine Tregs [15]. Although human Tregs unlike mice do not express substantial levels of CD73, it is reported that HIV infection is associated with the downregulation of CD73 on Tregs from those on ART but not in long-term non-progressors (LTNPs) [16].

In addition to the above-mentioned transcriptional factors, several microRNAs (miRNAs) are suggested to regulate CD73 expression by direct targeting of CD73 mRNA or indirect targeting of CD73 transcriptional factors [9]. Among these miRNAs, miR-30a, miR-30b, miR-187, and miR-193b have been reported to control CD73 expression in different cell lines by direct regulation of the CD73 gene [17–20]. On the other hand, miR-30a-e and miR-200c target SMAD2, a transcriptional activator of CD73, resulting in the downregulation of CD73 expression [21]. Similarly, miR-16 and miR-142-5p may downregulate CD73 expression by inhibiting SMAD-3, another transcriptional regulator of the CD73 gene [22,23].

In this study, we aimed to evaluate the surface expression of CD73 in both CD4^+^ and CD8^+^ T cells from different cohorts of HIV-infected individuals compared to healthy controls (HCs). We also measured CD73 at the intracellular and gene levels in CD8^+^ T cells of HIV-infected patients compared to HCs. Notably, we evaluated the functionality of CD73-expressing CD8^+^ T cells from HIV-infected and HCs. We further proposed that the elevated ATP in the plasma of HIV-infected individuals through enhanced expression of miRNA-30c-e downregulates CD73 expression in CD8^+^ T cells.

## Results

### Reduction of CD73 expressing T cells in HIV-infected individuals

**Fig 1:**
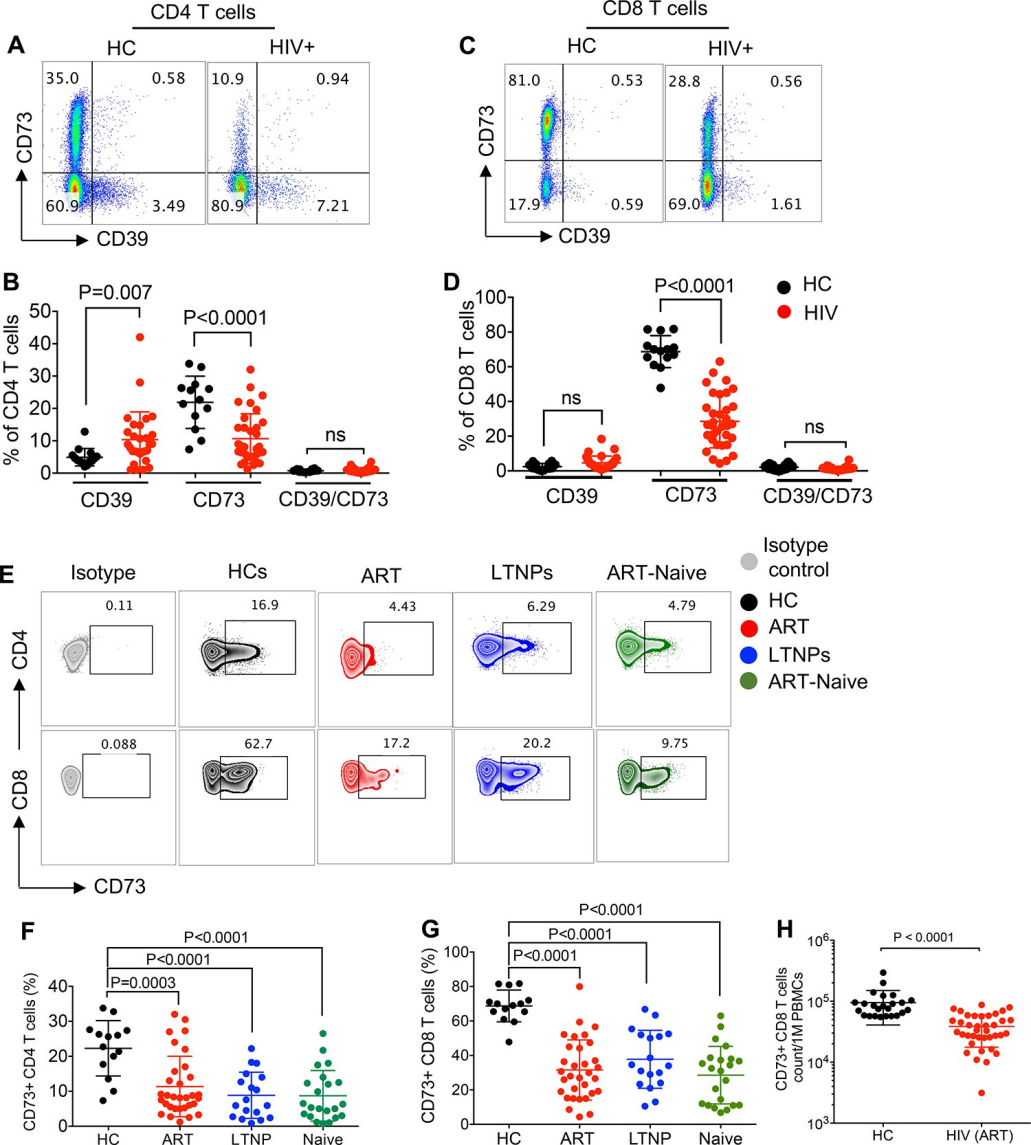
Lower frequency of CD73^+^ T cells in the peripheral blood of HIV-infected individuals. (**A**) Representative flow cytometry plots, and (**B**) Cumulative data showing percentages of CD39^+^ and CD73^+^ CD4^+^ T cells in healthy controls (HCs) versus HIV-infected individuals. (**C**) Representative flow cytometry plots, and (**D**) Cumulative data showing percentages of CD39^+^ and CD73^+^ CD8^+^ T cells in HCs versus HIV-infected individuals. (**E**) Representative flow cytometry plots, (**F**) Cumulative data showing percentages of CD73^+^CD4^+^ T cells, and (**G**) CD73^+^CD8^+^ T cells in HCs compared to HIV-infected individuals either on ART, ART-naive or LTNPs. (**H**) Cumulative data of total CD73^+^CD8^+^ T cells in PBMCs of HCs and HIV-infected individuals on ART. Each point represents one human subject either HC or HIV-infected individual. Data are obtained from multiple independent experiments. Each dot represents data from an individual human subject. Statistical analysis determined by the Mann-Whitney U-test (B, D, F-H) with significance indicated. ns: no significant.

Since CD39 and CD73 are two associated ectoenzymes, we first determined their frequency in PBMCs of HIV-infected individuals and HCs. In agreement with previous reports [24,25], we found that the percentages of CD39 expressing T cells were significantly elevated for CD4^+^ but not for CD8^+^ T cells in HIV-infected individuals compared to HCs (Fig 1A–1D; the gating strategy shown in S1A Fig). In contrast, we found percentages of CD73 expressing CD4^+^ and CD8^+^ T cells were significantly decreased in HIV-infected individuals compared to HCs (Fig 1A–1D), which is in agreement with a previous report [26]. Consequently, we decided to investigate the frequencies of CD73-expressing T cells in different HIV infected subgroups compared to HCs. We found that the frequency of CD73^+^CD4^+^ T cells was significantly decreased in individuals on ART, LTNPs, and ART-naïve individuals compared to HCs (Fig 1E and 1F). Similarly, the frequency of CD73 expressing CD8^+^ T cells was significantly lower in individuals on ART, LTNPs, and ART-naive individuals compared to HCs (Fig 1E and 1G). Since the reduction in the proportion of CD73 expressing T cells was more dominant among CD8^+^ versus CD4^+^ T cells, we quantified the total CD73^+^CD8^+^ T cells in PBMCs of HCs versus HIV-infected individuals on ART. These analyses revealed a significant decline in the total CD73^+^CD8^+^ T cells in HIV-infected individuals on ART compared to HCs (Fig 1H). It is worth mentioning that the reduction in CD73^+^ cells was beyond T cells and it appeared to be a general phenomenon in HIV infection as shown for CD3^-^ cells (S1B Fig). Thus, our results show higher percentages of CD39^+^ but lower CD73^+^ T cells in HIV-infected individuals compared to HCs.

### Lower CD73 intracellular protein and gene levels in CD8^+^ T cells from HIV-infected individuals

**Fig 2:**
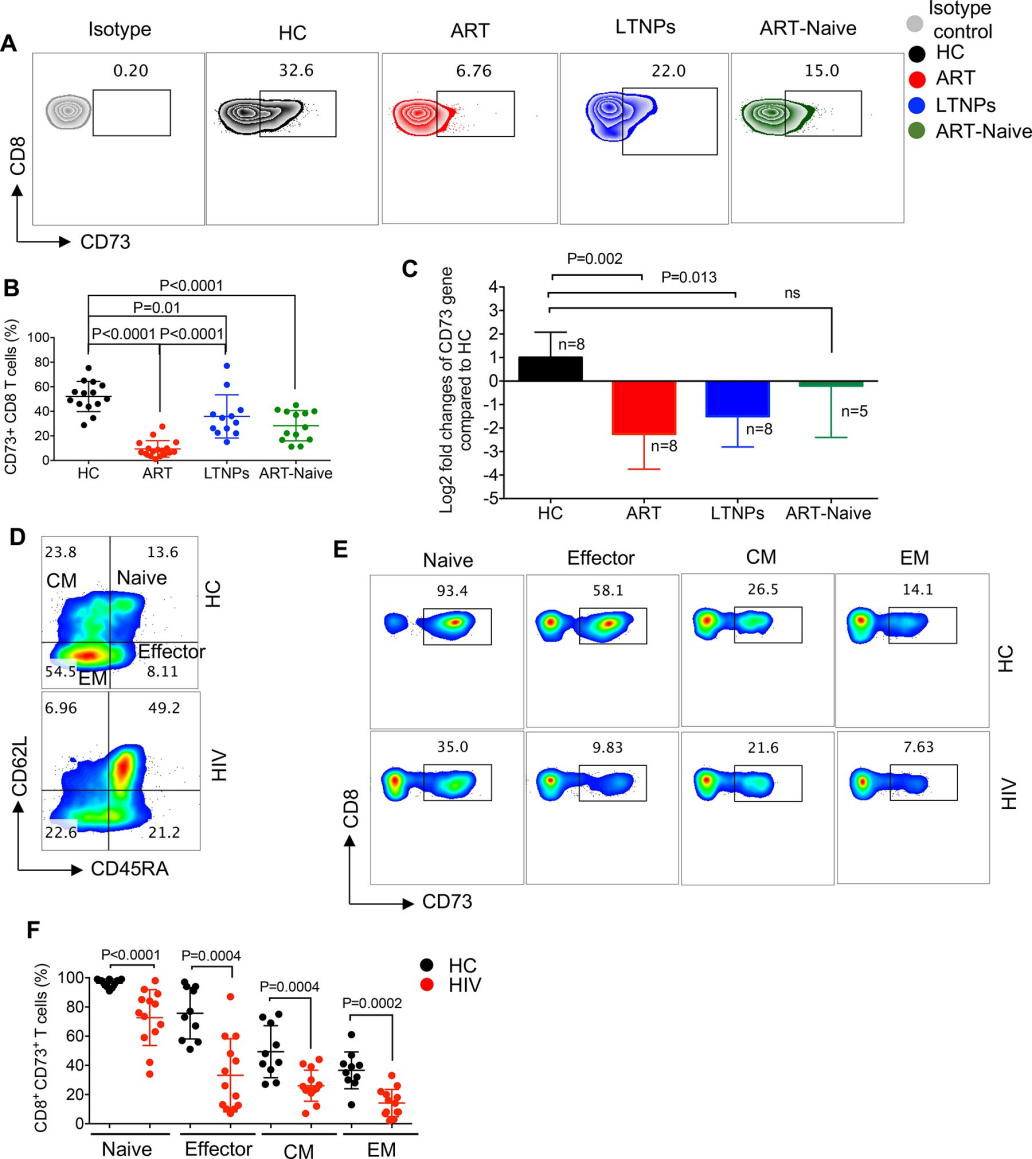
Global reduction in the frequency of CD73^+^CD8^+^ T cells in HIV-infected individuals. (**A**) Representative flow cytometry plots, and (**B**) Cumulative data showing intra-cellular expression level of CD73 in CD8^+^ T cells in HCs compared to HIV-infected individuals either on ART, ART-naive or LTNP. (**C**) Fold regulation of CD73 gene in CD8^+^ T cells of HIV-infected individuals (on ART, ART-naïve and LTNPs) relative to HCs quantified by qPCR. (**D**) Representative flow cytometry plot showing different subpopulations of CD8^+^ T cells in a HC versus a HIV-infected individual on ART. (**E**) Representative flow cytometry plots, and (**F**) Cumulative data showing expression of CD73 on different subsets of CD8^+^ T cells in HCs compared with HIV-infected individuals. Each point represents one human subject either HC or HIV-infected individual. Data are obtained from multiple independent experiments. Each dot represents data from an individual human subject. Statistical analysis determined by the Kruskal–Wallis test (B and C) and the Mann-Whitney U-test (F) with significance indicated. n: reflects the number of samples.

The focus of our study was CD8^+^ T cells, as the main anti-virus effector cells in HIV infection [27–29], thus, we did not purse CD4^+^ T cells in regards to the expression of CD73. To assess whether the lower frequency of CD73^+^CD8^+^ T cells results from the cell surface shedding of CD73 or a lower synthesis of CD73 in CD8^+^ T cells in HIV-infected individuals, we first measured the intracellular CD73 protein expression in CD8^+^ T cells. We observed that the expression of intracellular CD73 protein was significantly lower in CD8^+^ T cells of ART-naive, LTNPs, and individuals on ART compared to HCs (Fig 2A and 2B). Notably, the intracellular expression of CD73 was significantly lower in CD8^+^ T cells from patients on ART compared to LTNPs (Fig 2B). Also, we examined the CD73 gene expression by CD8^+^ T cells from different subpopulations of HIV-infected individuals compared to HCs. Interestingly we found lower CD73 gene expression in CD8^+^ T cells from HIV-infected individuals on ART and LTNPs compared to HCs (Fig 2C). Although CD73 gene expression was lower in ART-naïve CD8^+^ T cells, it didn’t reach a significance. Thus, our results showed a lower expression of CD73 in CD8^+^ T cells at the gene and protein levels in HIV-infected individuals. Moreover, we compared the levels of CD73 in the plasma, and we found lower CD73 plasma levels of CD73 in HIV-infected individuals compared to HCs (S1C Fig). These observations further confirmed that the reduced CD73 expression was not due to its shedding from T cells in HIV-infected individuals.

### The frequency of CD73^+^CD8^+^ T cells is reduced in HIV-infected individuals regardless of their differentiation status

Antigen stimulation results in the generation of effector and memory CD8^+^ T cells that are functionally and phenotypically different from naïve T cells [30–32]. Therefore, we questioned whether the decreased frequency of CD73^+^CD8^+^ T cells is associated with a specific CD8^+^ T cell subset in HIV-infected individuals. We first grouped CD8^+^ T cells into naïve (CD45RA^+^CD62L^+^), central memory (CM) (CD45RA^-^CD62L^+^), effector memory (EM) (CD45RA^-^ CD62L^-^), and effector cells (CD45RA^+^ CD62L^-^) (Fig 2D). Next, we compared the percentages of CD73^+^ cells in each subset of CD8^+^ T cells from HCs and HIV-infected individuals. We found that the frequency of CD73-expressing cells was significantly lower in all of the CD8^+^ T cell subsets from HIV-infected individuals compared to HCs (Fig 2E and 2F). These results show that the lower frequency of CD73-expressing cells is not restricted to any specific subset of CD8^+^ T cells but rather a general phenomenon in HIV-infected individuals.

### CD73 expressing CD8^+^ T cells exhibit a dysfunctional phenotype

**Fig 3:**
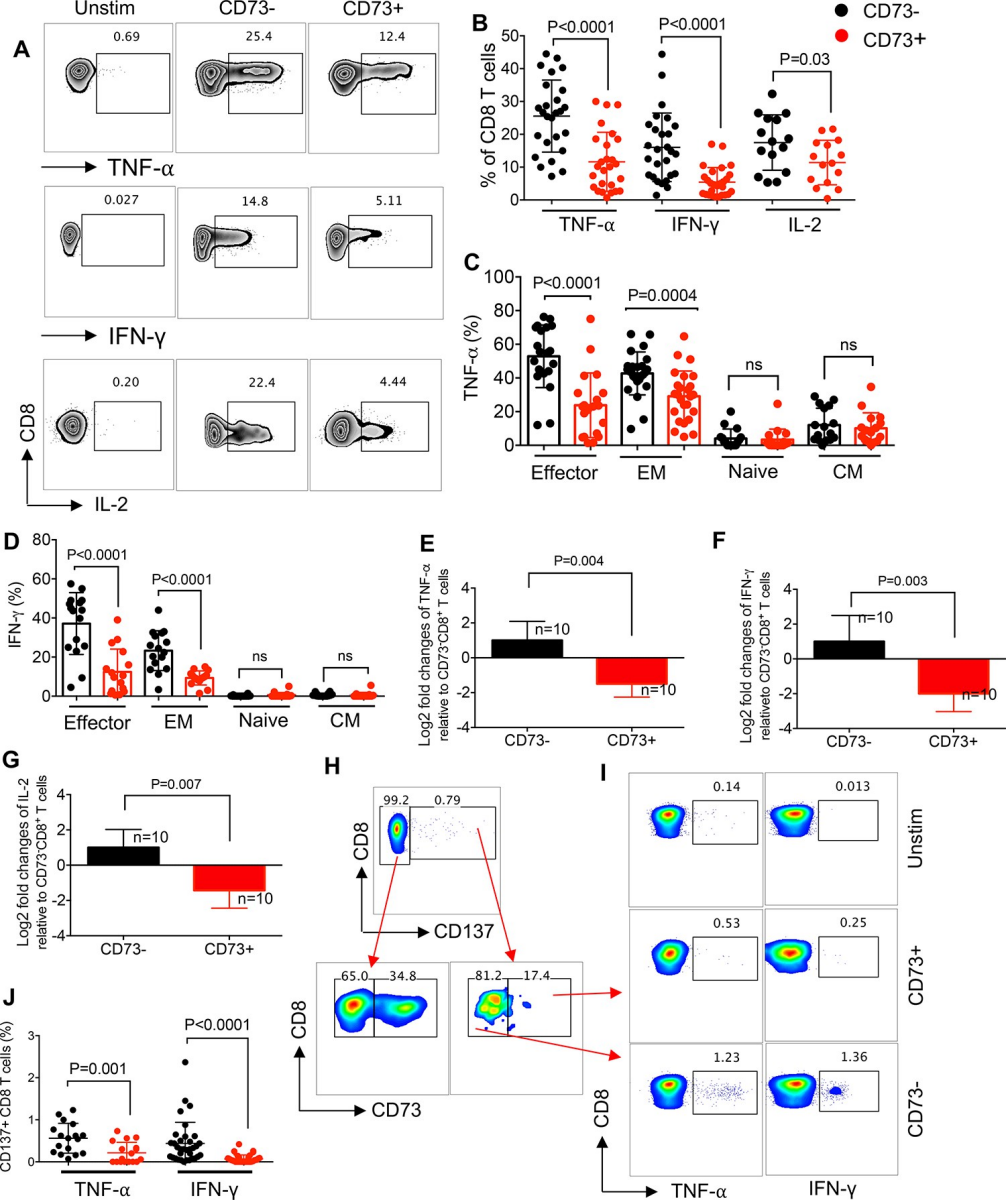
Impaired effector functions of CD73^+^CD8^+^ T cells compared to CD73^-^CD8^+^ T cells in PBMCs of HIV-infected individuals. (**A**) Representative flow cytometry plots, and (**B**) Cumulative data of TNF-α, IFN-γ, and IL-2 expression in CD73^+^ versus CD73^-^CD8 T cells upon stimulation of PBMCs from HIV-infected individuals with anti-CD3/CD28. (**C**) Cumulative data of TNF-α, and (**D**) IFN-γ expression in CD73^+^ versus CD73^-^ in different subsets of CD8^+^ T cells of HIV-infected individuals. (**E-G**) Fold regulation of TNF-α, IFN-γ, and IL-2 genes in CD73^+^CD8^+^ T cells relative to CD73^-^ CD8^+^ T cells of HIV-infected individuals quantified by qPCR. (**H**) Gating strategy and the representative plots showing CD73^+^ cells among CD137^+^ CD8^+^ T cells compared to CD137^-^CD8^+^ T cells, and (**I**) Cumulative data of TNF-α and IFN-γ expression in CD73^+^ versus CD73^-^ CD137^+^ CD8^+^ T cells upon stimulation of PBMCs from HIV-infected individuals with the Gag peptide pool. Each point represents one HIV-infected individual. Data are obtained from multiple independent experiments. Each dot represents data from an individual human subject. Statistical analysis determined by the Mann-Whitney U-test (B, C, D-G and J) with significance indicated. ns, no significant. n: reflects the number of samples.

Since we were limited with the quantity of cells from ART-naïve and LTNPs, our functional studies were performed on HIV-infected individuals on ART. To characterize the functionally of CD73^+^CD8^+^ T cell *in vitro*, PBMCs from HIV-infected individuals on ART were stimulated with anti-CD3/CD28 antibodies and cytokine production (TNF-α, IFN-γ, and IL-2) was assessed by intercellular cytokine staining in CD73^+^ versus CD73^-^CD8^+^ T cells. In contrast to what has been shown [26,33], we observed CD73^+^CD8^+^ T cells exhibited impaired functionality by expressing significantly lesser TNF-α, IFN-γ, and IL-2 compared to their negative counterparts (Fig 3A and 3B). The same phenotype was observed in CD73^+^CD8^+^ T cells from HCs in response to PBMC stimulation with anti-CD3/CD28 antibodies (S1D Fig). Since the other group had used phorbol-12-myristate-13-acetate (PMA) for cell stimulation [26], we stimulated PBMCs with PMA instead of anti-CD3/CD28 antibodies and once again we observed significantly lower cytokine expression by CD73^+^ versus CD73^-^ CD8^+^ T cells from HIV-infected individuals (S1E Fig). To investigate whether the decreased ability of cytokine production was consistent among different CD8^+^ T cell subpopulations, we examined the production of TNF-α and IFN-γ by various CD8^+^ T cell subsets. As we expected, effector and EM CD8^+^ T cells had the highest expression of cytokines compared to naïve and CM subsets. However, CD73 expressing cells had significantly lower levels of TNF-α and IFN-γ compared to their negative counterparts among both effector and EM subsets (Figs 3C and 3D, and S1F). Furthermore, we found that CD73^+^CD8^+^ T cells had significantly lower levels of TNF-α, IFN-γ, and IL-2 mRNA compared to their negative counterparts (Fig 3E–3G). Moreover, we assessed the frequency of antigen-specific cytokine-producing T cells among CD73^+^/CD73^-^ CD8^+^ T cells in HIV-infected individuals following PBMC stimulation with the HIV gag peptide pool. We identified antigen-specific CD8^+^ T cells based on the expression of CD137 as reported elsewhere [34,35] (Fig 3H). We found that antigen-responsive CD8^+^ T cells (CD137^+^) were mainly CD73^-^ (Fig 3H). Therefore, CD137^+^CD73^+^CD8^+^ T cells expressed significantly lower levels of TNF-α and IFN-γ compared to CD137^+^CD73^-^CD8^+^ T cells (Fig 3H and 3I). It is reported that CD73 acts as a receptor for different ligands such as tenascin C, fibronectin, laminin, and extracellular matrix proteins (ECM) [36–38]. Because some of these ligands (e.g. fibronectin) are also expressed on immune cells [39], we decided to determine if impaired functionality of CD73^+^CD8^+^ T cells was mediated via cell–cell interactions. To test this, we excluded the interaction of CD8^+^ T cells with other cells by isolating them from PBMC and measuring cytokine expression in isolated CD8^+^ T cells in HIV-infected individuals. However, we observed that even in the absence of other immune cell components of PBMCs, CD73^+^CD8^+^ T cells exhibited impaired functionality and expressed significantly lower TNF-α, IFN-γ, and IL-2 compared to CD73^-^ CD8^+^ T cells (S2A–S2C Fig). Finally, we measured the expression of activation markers (CD38 and HLA-DR) [40] that showed significantly lower levels of HLA-DR on CD73^+^CD8^+^ T cells compared to their negative counterparts, while a similar level of CD38 was expressed by CD73^+^/CD73^-^CD8^+^ T cells from HIV-infected individuals (S2D Fig). Therefore, our results show that CD73^+^CD8^+^ T cells have lower cytokine production capability compared to their negative counterparts.

### The expression of CD73 by CD8^+^ T cells is associated with impaired degranulation capacity

**Fig 4:**
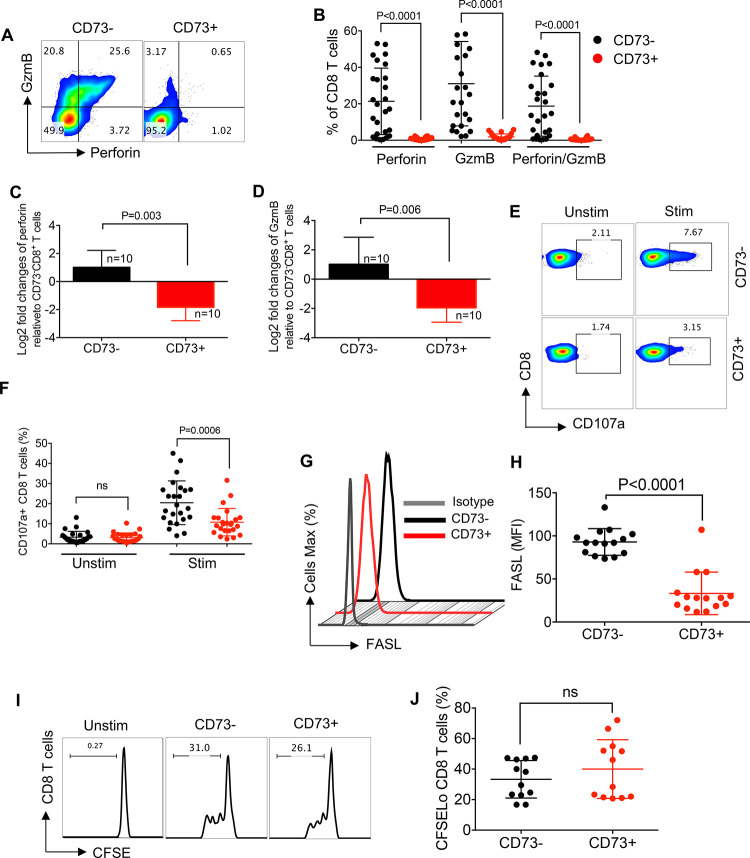
Impaired expression of cytolytic molecules in CD73^+^CD8^+^T cells. (**A**) Representative flow cytometry plots, and (**B**) cumulative data showing the expression of perforin, GzmB and perforin/GzmB in CD73^+^ versus CD73^-^ CD8^+^ T cells. (**C**) Fold regulation of perforin gene, and (**D**) GzmB gene in CD73^+^ relative to CD73^-^CD8^+^ T cells in HIV-infected individuals quantified by qPCR. (**E**) Representative flow cytometry plots of CD107a, and (**F**) cumulative data of CD107a in CD73^-^ versus CD73^+^ CD8^+^ T cells pre- and post-stimulation with anti-CD3/CD28 antibodies. (**G**) Representative histogram plots, and (**H**) cumulative data of FASL expression as measured by the mean fluorescence intensity (MFI) in CD73^-^ versus CD73^+^ CD8^+^ T cells on HIV-infected individuals. (**I**) Representative flow cytometry plots, and (**J**) cumulative data of CD73^+^ versus CD73^-^ CD8^+^ T cells proliferation measured by CFSE after 3 days of stimulation with anti-CD3/CD28 antibodies. Each point represents one HIV-infected individual. Data are obtained from multiple independent experiments. Each dot represents data from an individual human subject. Statistical analysis determined by the Mann-Whitney U-test (B, C, D, F, H and J) with significance indicated. ns: no significant. n: reflects the number of samples.

To determine the impact of CD73 expression on the cytotoxic ability of CD8^+^ T cells, we compared perforin and GzmB content of CD73^+^ versus CD73^-^CD8^+^ T cells from HIV-infected individuals. We observed that the expression of CD73 by CD8^+^ T cells was associated with a significant reduction in the expression of both perforin and GzmB expression (Fig 4A and 4B). We also observed significantly lower perforin and GzmB expression at the gene levels in CD73^+^CD8^+^ T cells compared to their CD73- counterparts (Fig 4C and 4D). We also investigated the degranulation capacity of CD73^+^ versus CD73^-^CD8^+^ T cells by measuring the expression of the lysosomal-associated membrane protein 1 (LAMP1; or CD107a) [41]. PBMCs from HIV-infected individuals were stained with an anti-CD107a antibody before stimulation with anti-CD3/CD28 antibodies for 6 h. We found that the expression of CD107a was not significantly different between CD73^+^ and CD73^-^CD8^+^ T cells in the absence of TCR stimulation (Fig 4E and 4F). However, upon TCR stimulation, CD73^+^CD8^+^ T cells exhibited impaired degranulation compared to CD73^-^ CD8^+^ T cells (Fig 4E and 4F). Since CD8^+^ T cell-mediated cytotoxicity can also occur via the interaction of FasL: Fas on target cells [42,43], we examined the expression of FasL on CD73^+^ CD8^+^ T cells, which showed lower expression of FasL compared to CD73^-^CD8^+^ T cells (Fig 4G and 4H). However, we did not find any difference between the proliferative capacity of CD73^+^ versus CD73^-^CD8^+^ T cells (Fig 4I and 4J). These results show that CD73-expressing CD8^+^ T cells exhibit an impaired cytotoxic phenotype compared to their negative siblings within the same individual.

### CD73^+^CD8^+^ T cells exhibit a greater migratory capability

**Fig 5:**
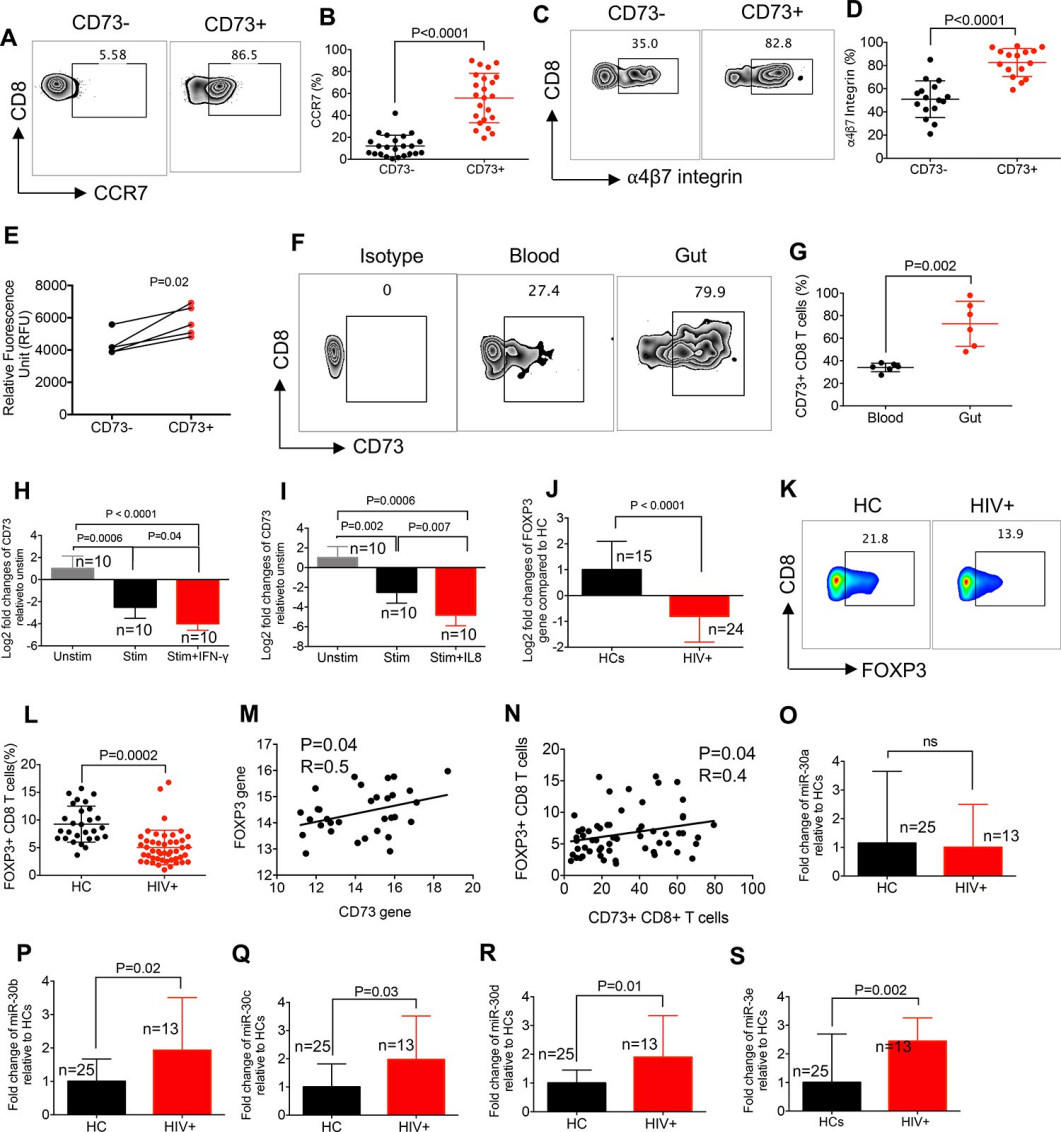
Upregulation of miRNA30b-30e in CD8^+^ T cells of HIV-infected individuals. (**A**) Representative flow cytometry plots, and (**B**) cumulative data of CCR7 expression on CD73^-^ versus CD73^+^ CD8^+^ T cells. (**C**) Representative flow cytometry plots, and (**D**) cumulative data of ⍺4β7 integrin expression on CD73^-^ versus CD73^+^ CD8^+^ T cells. (**E**) Cumulative results of migratory ability of effector CD8^+^CD73^-^ versus CD8^+^CD73^+^ T cells as measured by RFU. (**F**) Representative flow cytometry plots, and (**G**) cumulative data of the percentage of CD73 expressing CD8^+^ T cells in the peripheral blood and the gut tissue of mice. (**H**) Fold regulation of CD73 gene in isolated CD8^+^ T cells either unstimulated (unstim) or stimulated (stim) with anti-CD3/CD28 antibodies in the presence or absence of IFN-γ (100 ng/mL), and (**I**) IL-8 (100 ng/mL). (**J**) Fold regulation of FOXP3 gene in CD8^+^ T cells isolated from PMBCs of HIV-infected individuals relative to HCs quantified by qPCR. (**K**) Representative flow cytometry plots, and (**L**) cumulative data of FOXP3 expression in CD8^+^ T cells from HCs vs HIV-infected individuals. (**M**) Cumulative data of the correlation between the CD73 gene and the FOXP3 gene in CD8^+^ T cells of HIV-infected individuals and HCs. (**N**) Cumulative data of the correlation between the cell surface CD73 and FOXP3 expression in CD8^+^ T cells of HIV-infected individuals and HCs. (**O**) Fold change of miRNA-30a, (**P**) miRNA-30b, (**Q**) miRNA-30c, (**R**) miRNA-30d, and (**S**) miRNA30-e in CD8^+^ T cells of HIV-infected individuals relative to HCs quantified by qPCR. Each dot represents data from an individual human subject. Each dot represents data from an individual human subject. Statistical analysis determined by the Mann-Whitney U-test (B, D, E, G, J, L, and O-S), Kruskal–Wallis test (H and I), and by linear regression analysis (M and N) with significance indicated. ns: no significant. “n” reflects the number of samples.

It is reported that CD73 is involved in CD8^+^ T cells adhesion to endothelial cells and lymphocyte extravasation in an LFA-1-dependent mechanism [8]. Moreover, the role of CD73 in CD4^+^ T cells for the efficient entry into the central nervous system during the development of experimental autoimmune encephalomyelitis has been documented [44]. Also, CD73 participates in the migration of lymphocytes via the afferent lymphatic vessels and intestinal lymphoid tissues [45,46]. To delineate the potential role of CD73 in T cell trafficking, we compared the expression of CCR7 and ⍺4β7 integrin on CD73^+^ and CD73^-^ CD8^+^ T cells. We found that CD73^+^CD8^+^ T cells expressed significantly higher levels of both CCR7 and ⍺4β7 integrin compared to their negative counterparts (Fig 5A–5D). These observations suggest a differential capability of trafficking for CD73^+^CD8^+^ T cells since these molecules are associated with T cell trafficking to secondary lymphoid organs and the gut [47,48]. This hypothesis was further supported when we observed significantly a greater migratory capacity for the effector CD73^+^ versus CD73^-^CD8^+^ T cells *in vitro* (Fig 5E). We further confirmed this concept by measuring the expression of CD73 in CD8^+^ T cells in the gut tissue versus the peripheral blood in mice. As shown in Fig 5F and 5G, we found a significant abundance of CD73^+^CD8^+^T cells in the gut tissues compared to the peripheral blood in mice. These results may support the concept of a higher capacity of CD73^+^CD8^+^ T cells to migrate to lymph nodes and gut-associated lymphoid tissues (GALT), the sites that harbour HIV reservoirs [49]. As proof of concept, we investigated the frequency of CD73-expressing CD8^+^ T cells in the cerebrospinal fluids (CSF) of relapsing-remitting multiple sclerosis (MS) patients at the time of disease remission versus relapse. Interestingly, we found a significantly higher abundance of CD73^+^CD8^+^ T cells in the CSF of MS patients at time of relapse (S2E and S2F Fig). These observations suggest that CD73^+^CD8^+^ T cells might exhibit a greater trafficking capacity.

### Lower levels of CD73 corresponds with FOXP3 expression in CD8^+^ T cells in HIV-infected individuals

We then decided to investigate the underlying mechanism(s) associated with a lower frequency of CD73-expressing CD8^+^ T cells in HIV-infected individuals. It is reported that chronic immune activation due to persistent HIV antigens results in increased plasma levels of several cytokines in the plasma, such as IL-2, IL-8, IL-10, IL-15, IL-16, IFN-α, IFN-γ, TNF-α, and TGF-β [50–54]. Thus, we investigated the effect of these cytokines on the expression of the CD73 gene in isolated CD8^+^ T cells from HCs following stimulation with anti-CD3/CD28 antibodies *in vitro*. Interestingly, we observed that T cell activation, in general, downregulates CD73 at the mRNA level (S3A–S3G Fig). While the addition of IL-2, IL-15, IL-16, TNF-α, and IFN-α had no synergistic effect on the downregulation of CD73 mRNA, the addition of IL-10 and TGF-β upregulated the expression of CD73 in CD8^+^ T cells (S3A–S3G Fig). In contrast, we observed a synergistic effect for IFN-γ and IL-8 cytokines and T cell stimulation, resulting in a significant downregulation of CD73 mRNA in CD8^+^ T cells (Fig 5H and 5I).

Next, we measured the expression level of several transcription factors and miRNAs that are reported to regulate CD73 expression in CD8^+^ T cells. For example, it is well recognized that FOXP3 through permissive H3 modification upregulates the expression of CD73 in Tregs [15]. Thus, we quantified the expression of the FOXP3 gene in CD8^+^ T cells of HIV-infected individuals compared to HCs. We found significantly lower FOXP3 expression at the gene and protein levels in CD8^+^ T cells of HIV-infected individuals on ART versus HCs (Fig 5J–5L). Also, a lower CD73 expression in Tergs of HIV-infected individuals has been reported [16]. These observations were further supported by the positive correlation between the expression of FOXP3 and CD73 at the gene and protein levels in CD8^+^ T cells of both HIV-infected individuals and HCs (Fig 5M and 5N).

### The High plasma ATP downregulates CD73 in CD8^+^ T cells through the upregulation of miR-30c-30e

Since the role of miRNAs in regulating CD73 expression in different cell lines has been suggested [9], we aimed to determine whether miRNAs regulate the expression of CD73 in CD8^+^ T cells. It is shown that the miR-30 family downregulates CD73 expression by direct binding to its promoter [55], or by the downregulation of SMAD2 expression [21]. Thus, we analyzed the expression of miR-30 family members in CD8^+^ T cells isolated from both HIV-infected and healthy individuals. We found that the expression of miR-30a was not significantly different between HCs vs HIV-infected individuals (Fig 5O). However, we observed a significantly higher expression of miR-30b-e in CD8^+^ T cells isolated from HIV-infected individuals compared to HCs (Fig 5P–5S). Also, we quantified the expression of a wide range of other miRNAs (e.g. miR-193, miR-187, miR-422, miR-146, miR-130a, miR142-3p, miR142-5p) in CD8^+^ T cells of HIV-infected versus healthy individuals but we did not find any significant difference.

**Fig 6:**
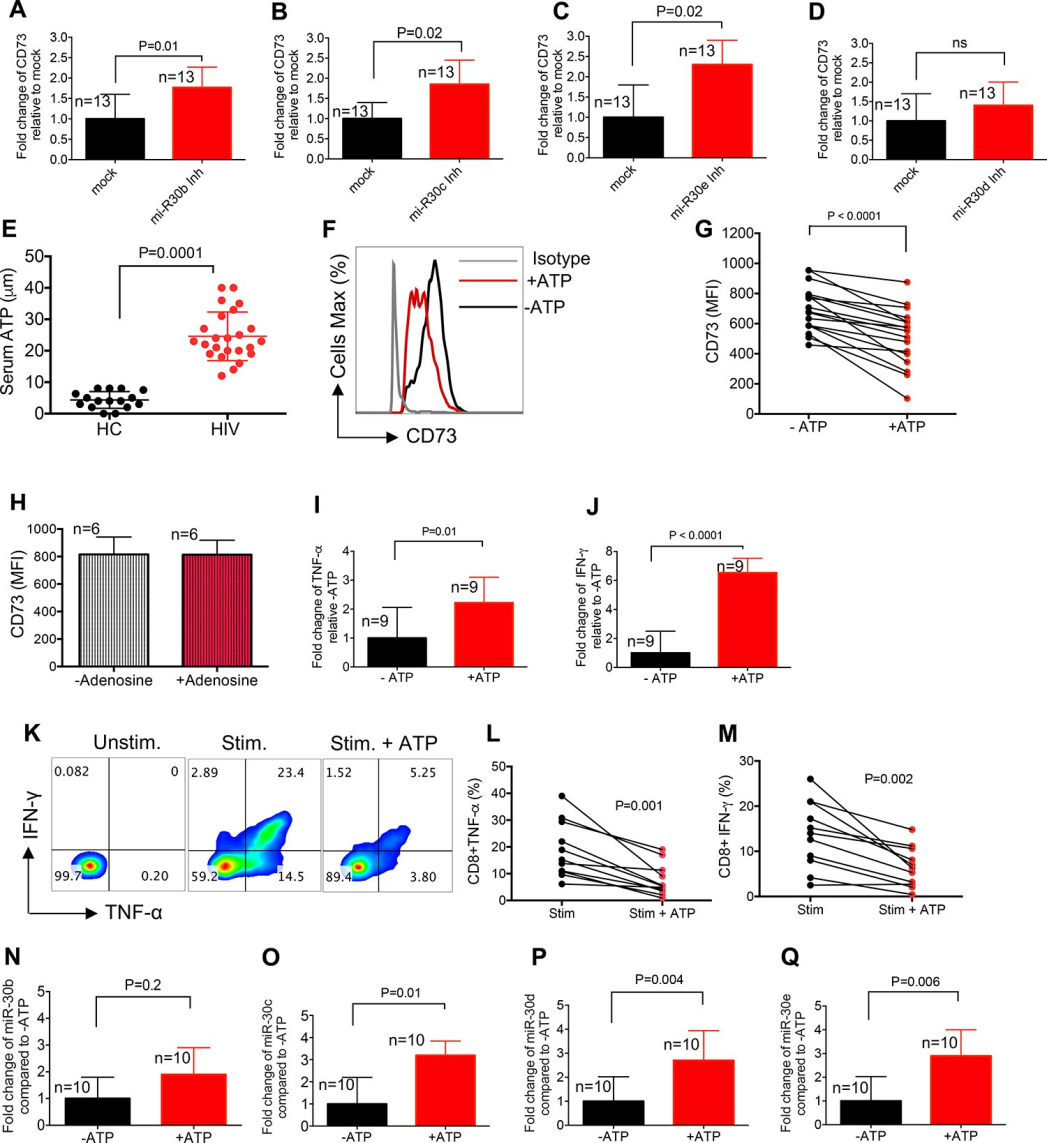
Higher levels of the plasma ATP may upregulate the expression of miRNA30b-30e in HIV-infected individuals. Fold regulation of the CD73 gene in isolated CD8^+^ T cells after treatment with (**A**) the miR-30b, (**B**) miR-30c, (**C**) miR-30d, and (**D**) miR-30e inhibitors. (**E**) Quantification of ATP levels in the plasma of HIV infected individuals versus HCs using ATP light kit. (**F**) Representative histogram plots of CD73 expression in CD8^+^ T cells with/without treatment with ATP (25 μM) for 72 hr. (**G**) Cumulative data showing MFI of CD73 in CD8^+^ T cells after their treatment with ATP. (**H**) Cumulative data showing MFI for CD73 in CD8^+^ T cells treated with or without adenosine (10 μM) for 72 hr. (**I**) Fold change of TNF-α, and (**J**) IFN-γ genes in CD8^+^ T cells treated with or without ATP (25 μM) for 72 hr. (**K**) Representative flow cytometry plots, and (**L**) cumulative data for TNF-α, and (**M**) IFN-γ expression in CD8^+^ T cells stimulated (stim) with anti-CD3/CD28 antibodies overnight without or with ATP (25 μM). Unstimulated (Unstim) as negative control. (**N**) Fold change of miR-30b, (**O**) miR-30c, (**P**) miR-30d, and (**Q**) miR-30e in isolated CD8^+^ T cells after treatment with ATP (25 μM) for 2 hr. Each dot represents data from an individual human subject. Statistical analysis determined by the Mann-Whitney U-test (A-E, H-J, and N-Q), Wilcoxon matched-pairs test (G, L and M) with significance indicated. ns: no significant. n: reflects the number of samples.

To investigate whether overexpression of miR-30b-30e results in the downregulation of CD73 in CD8^+^ T cells of HIV-infected individuals, we inhibited these miRNAs using miRNA inhibitors and quantified the expression of CD73 at the gene level [56]. First, we confirmed that miRNA inhibitors significantly downregulated the expression of our target miRNAs (S3H–S3K Fig). We found that inhibition of miR-30b,30c, and 30e resulted in the upregulation of the CD73 gene in CD8^+^ T cells (Fig 6A–6C). However, this was not the case when the miR-30d was inhibited (Fig 6D). These observations suggest that the upregulation of miR-30b, 30c, and 30e may contribute to the decreased expression of CD73 in CD8^+^ T cells in HIV-infected individuals.

**Fig 7:**
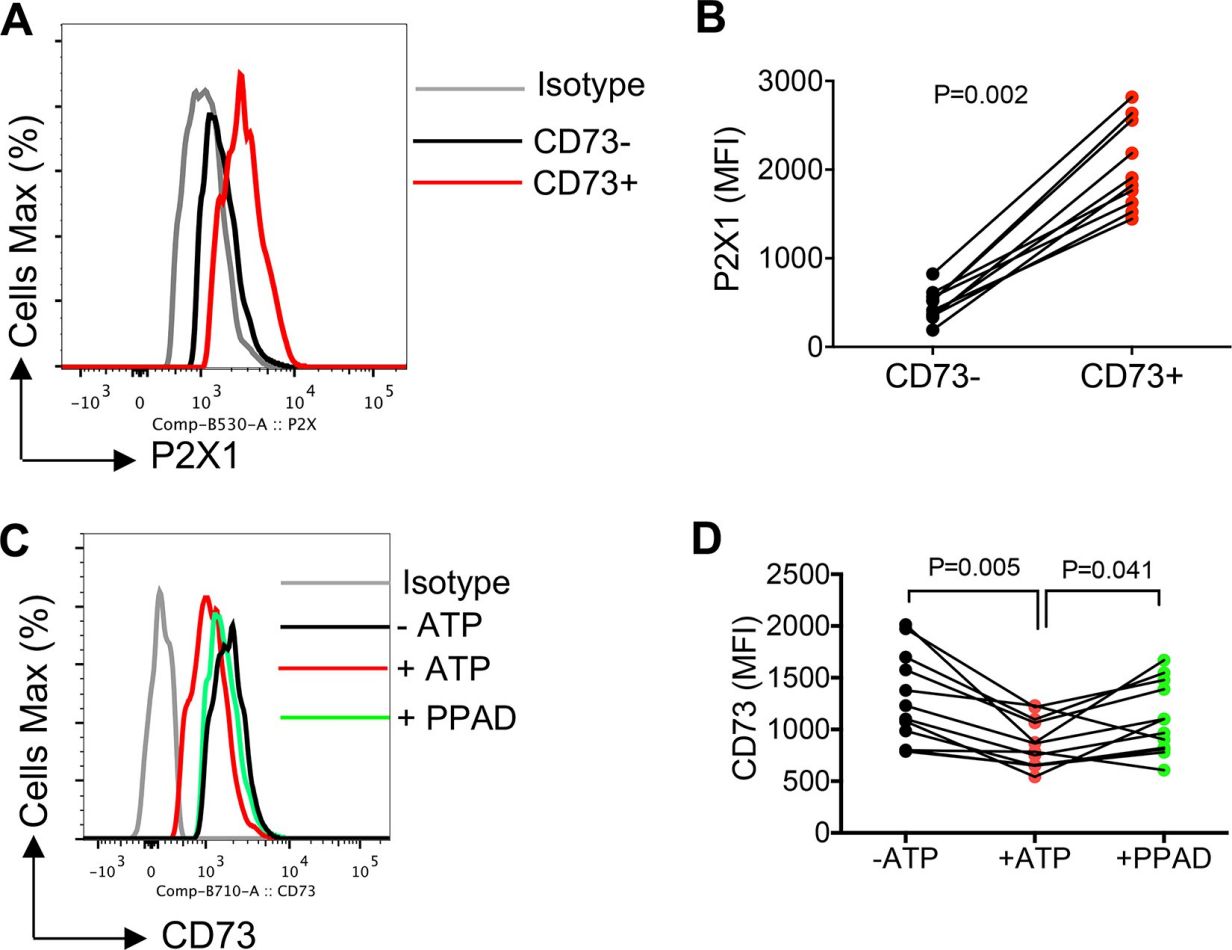
P2X1 is strongly expressed on CD73^+^CD8^+^ T cells and its inhibition partially restores the potential effects of ATP. (**A**) Representative histogram plots, and (**B**) cumulative data showing P2X1 expression in CD73^-^/CD73^+^ CD8^+^ T cells. (**C**) Representative histogram plots, and (**D**) cumulative data of CD73 expression with/without treatment with ATP (25 μM) and PPAD (20 μM) for 48 hr. Statistical analysis determined by the Mann-Whitney U-test (B), Wilcoxon matched-pairs test (D) with significance indicated. Each dot represents results from a study subject.

To provide an insight into the potential mechanism(s) linked to the upregulation of miR30b-e in CD8^+^ T cells, we quantified the plasma ATP in HIV-infected individuals. In general, CD73 acts in tandem with CD39 on the cell surface to convert ATP to AMP and then adenosine [5]. Thus, we speculated whether decreased CD73 results in the accumulation of upstream ATP in the plasma of HIV-infected individuals. Interestingly, we observed significantly higher levels of ATP in the plasma of HIV-infected individuals compared to HCs (Fig 6E). We further determined the effect of increased ATP on CD73 expression in CD8^+^ T cells. For this, we treated PMBCs from HIV-infected individuals with ATP (25 μM) for 72 hours and then examined the expression of CD73 in CD8^+^ T cells. Interestingly, we found that ATP treatment significantly decreased the expression of CD73 in CD8^+^ T cells (Fig 6F and 6G). However, treatment of PBMCs with adenosine (10 μM) for 72 hours did not change the expression of CD73 in CD8^+^ T cells (Fig 6H). Next, we investigated the effects of ATP on cytokine production at the gene and protein levels. We found that ATP (25 μM) treatment for 2 hours significantly increased TNF-α and IFN-γ genes (Fig 6I and 6J), however, prolonged treatment with ATP (25 μM, 18 hours) resulted in a significant reduction in the same cytokines at the protein level when PBMCs were stimulated with anti-CD3/CD28 (Fig 6K–6M). To determine if the effect of ATP on CD73 was mediated through the elevation of the miR-30 family, we measured the levels of miR-30b-e in CD8^+^ T cells when treated with ATP. We found that ATP significantly upregulated the expression of miR-30c-e in CD8^+^ T cells (Fig 6N–6Q). To determine whether ATP receptors are involved in the process, we assessed the expression of ATP receptors. First, we assessed the expression of ATP receptors in murine CD8^+^ T cells which showed P2X was highly expressed on CD73^+^ CD8^+^ T cells. Next, we examined and found significant elevation of P2X1/T2RX1 or purinergic receptor P2X [57] on CD73^+^CD8^+^ T cells in human PBMCs (Fig 7A and 7B). Finally, we investigated the role of P2X in ATP-mediated downregulation of CD73 involving antagonism with PPAD [58]. We found that P2X inhibition significantly reversed CD73 downregulation by ATP after 48 hr incubation (Fig 7C and 7D). Therefore, our results suggest that the increased ATP level in the plasma of HIV-infected individuals might act through purinergic receptor and modulation of miRNAs resulting in the downregulation of CD73 in CD8^+^ T cells.

## Discussion

In this report, we show significantly lower frequency of CD73 expressing CD4^+^ and CD8^+^ T cells in HIV-infected individuals, which is in agreement with another report [26]. Our results show that the proportion of CD73 expressing T cells is consistently lower among both CD4^+^ and CD8^+^ T cells of all HIV-infected subgroups (e.g. LTNPs, ART-naïve, and those on ART). Surprisingly, this decline was more pronounced in HIV-infected individuals on ART. This might be related to the chronicity of the infection or the effects of the ART. It has been reported that CD73 is downregulated upon CD8^+^ T cell activation [5]. However, our results demonstrate a reduction in CD73 expressing cells in different subsets of CD8^+^ T cells suggesting that the decreased CD73 is a general feature of CD8^+^ T cell regardless of their phenotype (e.g. memory, effector, or naïve) in HIV infection.

CD73 is a heterodimer anchored to the plasma membrane through a GPI-anchor, which is sensitive to hydrolysis by endogenous phospholipases [59,60]. Both membrane-bound and soluble forms of CD73 have a similar affinity for AMP and exhibit similar AMPase activity [59], which can be inhibited by the adenosine 5′-(α,β-methylene)-diphosphate (APCP), a CD73 inhibitor [61]. Thus, we speculated whether decreased frequency of CD73 expressing CD8^+^ T cells results from its shedding from the cell surface. However, our results refuted this concept as we observed lower soluble CD73 in the plasma samples and reduced expression of CD73 at the gene level in CD8^+^ T cells of HIV-infected individuals, which supports a lower CD73 synthesis.

Our further studies to characterize the functionality of CD73^+^CD8^+^ T cells revealed that these cells exhibit impaired effector functions including lower inflammatory cytokines production, cytolytic molecules expression and degranulation compared to their negative counterparts following *in vitro* global (e.g. anti-CD3/CD28 and/or PMA) or HIV Gag peptide pool stimulation. It is worth mentioning that CD73^+^CD8^+^ T cells in HCs had similar effector functions compared to CD73^-^CD8^+^ T cells. Our further studies showed that the expression of CD73 was not restricted to a subset of T cells but expressed by different CD8^+^ T cell subsets with the highest expression among naïve followed by effector T cells. This led us to examine the functionality of CD73^+^ effector and EM CD8^+^ T cells, which once again confirmed their impaired effector functions. Therefore, we found that CD73^+^CD8^+^ T cells exhibit an impaired phenotype regardless of their differentiation status. We and others have shown that terminally impaired CD8^+^ T cells express a higher amount of mRNA for cytokines despite the severe defect in their cytokine production capacity in HIV-infected individuals [62,63]. Nevertheless, we noted lower expression of cytokines at the mRNA level in CD73^+^CD8^+^ T cells, which suggests CD73^+^CD8^+^ T cells have impaired pro-inflammatory cytokine production capability even at the gene level. This proposes that CD73 may suppress intrinsic signaling pathways through adenosine production [64]. These results are in disagreement with a previous report claiming that CD73^+^ EM CD8^+^ T cells had more IL-2 and TNF-α production following stimulation with PMA and HIV peptides, respectively [26]. Another group has shown that CM, EM, and effector CD73^+^CD8^+^ T cells produce significantly higher IL-2 upon TCR stimulation compared to their negative counterpart in patients with acute myeloid leukemia (AML) [33]. They also showed that antigen-specific CD73^+^CD8^+^ T cells produce higher levels of TNF-α, IFN-γ, and IL-2 in response to WT-1, a tumor-associated antigen [33]. This discrepancy might be related to different and questionable protocols used for T cell stimulation *in vitro*. Interestingly, in one of these studies, PBMCs were stimulated overnight with the PMA in the presence of a Golgi blocker. Since the recommended time for the treatment of cells with the Golgi blocker is 4–6 hr, it’s unclear whether overnight culture resulted in increased T cell toxicity. The recommended concentration of Golgi blocker is 1 μg/ml but this group has used (10 g/ml) [26]. Thus, we analyzed the toxicity of the Golgi blocker at 1 μg/ml and 10 μg/ml overnight on stimulated PBMCs with PMA. As anticipated, we found > 80% and 100% toxicity, respectively. The other group had expanded CD8^+^ T cells from AML patients in the presence of exogenous IL-2 for 6 days before conducting functional analysis [33]. In contrast, we performed our functional studies on freshly isolated PBMCs from HIV-infected individuals and HCs without any additional *in vitro* manipulation. Therefore, we believe our results provide an insight into the functionality of CD73^+^CD8^+^ T cells. Besides, we examined the expression of cytokines at the mRNA level and other effector functions such as cytolytic molecules expression and degranulation capacity in T cells. These observations altogether confirm an impaired effector phenotype in CD73^+^CD8^+^T cells.

The impact of CD73 downregulation in the context of HIV infection is not well understood. There is a possibility that the lower CD73 expression on T cells impairs the conversion process of AMP to the anti-inflammatory modulator, adenosine. As such lower adenosine may facilitate the observed chronic inflammation in HIV-infected patients [4]. In turn, chronic immune activation is accompanied by increased levels of multiple cytokines and ATP in the plasma of HIV-infected individuals [65,66]. Interestingly, we found that the addition of IFN-γ and IL-8 downregulate CD73 expression in CD8^+^ T cells once stimulated with anti-CD3/CD28 antibodies *in vitro*. Similarly, we observed exogenous ATP via purinergic receptor, P2X, downregulates CD73 expression in CD8^+^ T cells when examined *in vitro*, however, this was not the case for adenosine. These observations suggest that higher plasma IFN-γ and IL-8 might be linked to the downregulation of CD73 expression from HIV-infected individuals, both of which are elevated in the plasma of HIV-infected individuals [67,68]. Moreover, we found that a prolonged exposure of PBMCs to ATP inhibits cytokine expression in CD8^+^ T cells. These observations suggest that the elevated plasma ATP level in HIV infection may contribute to CD8^+^ T cell exhaustion [62]. Notably, we found that increased ATP facilitates the overexpression of minR-30b, 30c, 30d and 30e in CD8^+^ T cells. Therefore, our results imply that the increased ATP level in the plasma of HIV-infected individuals via enhanced miR-30c, 30d, and 30e downregulates the expression of CD73 in CD8^+^ T cells.

The major unanswered question is that what are the biological properties of CD73^+^CD8^+^ T cells and why they have minimal effector functions? First of all, as we have seen that CD73 is highly expressed on naïve CD8^+^ T cells. This suggests that CD73 downregulation may be essential to prevent autocrine adenosine signaling during CD8^+^ T cell activation, which is necessary for the transition of naïve to effector cells [5]. However, our observations show that CD73 is not restricted to naïve but also effector and memory CD8^+^ T cells. Thus, we believe that CD73 is required for CD8^+^ T cells migration into the tissue. In CD73 knockout mice, migration of lymphocytes to the draining lymph nodes via afferent lymphatic vessels is impaired [45]. Also, CD73 promotes the binding of lymphocytes to endothelial cells through an LFA1-dependent mechanism [8]. Moreover, the expression of CD73 on CD4^+^ T cells is required for the efficient entry into the central nervous system in the experimental autoimmune encephalomyelitis (EAE) model [44]. Consistent with these findings, it has been shown that individuals with HIV infection have a reduced risk of developing MS and/or experience lower relapse rates [69]. In agreement, we found a significant increase in the presence of CD73^+^CD8^+^ T cells in the CSF of MS patients. This was further supported by the increased migratory ability of CD73^+^CD8^+^ T cells *in vitro*. Moreover, we found that CD73 was highly co-expressed with CCR7 and ⍺4β7 integrin in CD8^+^ T cells, markers associated with T cell migration to secondary lymphoid tissues [70,71]. It is worth noting that one of the major barriers for HIV eradication is the presence of HIV reservoirs in different tissues, such as the spleen, lymph nodes (LNs), gut-associated lymphoid tissue (GALT), and the central nervous system (CNS) [49]. We speculate that the lower frequency of CD73^+^CD8^+^ T cells in HIV-infected individuals might account for the deficient access of CD8^+^ T cells to target tissues that harbor viral reservoirs. However, further studies are required to examine this possibility.

CD73 works with CD39 to convert ATP to immunosuppressive adenosine. Consequently, lower CD73 expression on T cells and other immune cells [26,72] in the context of HIV may lead to the accumulation of ATP in the plasma. As such, this scenario establishes a vicious cycle through which ATP decreases the expression of CD73, further contributing to the accumulation of ATP in the plasma of HIV-infected individuals. The same phenomenon may occur in other pathological conditions upon the elevation of plasma ATP.

Taken together, our findings demonstrate a lower expression of CD73 on T cells in HIV-infected individuals as reported in COVID-19 infected individuals [73]. However, further studies are required to determine the role of CD73 on T cells in other viral infections and cancer. These studies will assist us to better understand the mechanism (s) associated with the lower expression of CD73 on T cells and how it can be prevented. Therefore, such investigations may enable us to develop therapeutic interventions that reverse CD73 downregulation on CD8^+^ T cells in HIV infection and beyond. In contrast, our results suggest that targeting CD73 (e.g. anti-CD73 antibody) should be considered as a therapeutic approach in MS and other T cell-associated inflammatory conditions. However, this concept merits further investigations.

We are aware of multiple study limitations such as the lack of access to biopsies from HCs and HIV-infected individuals to determine if there are differences in the frequency of tissue resident CD8^+^CD73^+^ T cells. Although we have proposed elevated levels of ATP as a potential mechanism for CD73 downregulation, there is a possibility that lower CD73 leads to the accumulation of ATP in the plasma of HIV-infected individuals. This is similar to the chicken and egg casualty dilemma, which comes first: the elevated ATP or CD73 downregulation. Regardless of this point our results support a novel mechanism for CD73 downregulation via miRNAs. However, further studies are needed to investigate the role of ATP once the respective miRNAs are knocked out of CD8^+^ T cells. Also, CSF collection from HCs is not common and such samples are highly informative to compare the frequency of CD8^+^CD73^+^ T cells in the CFS of HCs compared to MS patients. Moreover, we were limited with the number of MS patients, therefore, performing similar studies on a larger cohort is required. Furthermore, comparing the effector functions of CD73^+^CD8^+^ T cells in the peripheral blood versus tissues is essential to determine if following migration these cells gain any functional properties. Finally, due to unavailability of flow cytometry based antibodies we were unable to measure the expression level of other ATP receptors in CD73^+^ T cells, which merits further investigations.

## Methods

### Ethics statement

This study was approved by the Research Ethics Board at the University of Alberta (protocol # Pro000046064 and Pro000070528). A written informed consent form was obtained from all study participants. Similarly, animal studies were approved by the Research Ethics Board at the University of Alberta (protocol # AUP00001021).

### Study population

124 human subjects were recruited for our studies (S1 Table) including: 1) 26 HIV-infected but antiretroviral therapy (ART)-naive individuals; 2) 63 HIV-infected individuals on ART (ART); and 3) 13 Long-term non-progressors (LTNPs), who had a plasma viral load <10,000 copies/ml, CD4 count >400 and were not on ART as defined in our previous reports [25,34,74]. We also recruited 22 HCs who were HIV, hepatitis B and C viruses seronegative. Apart from Figs 1 and 2A–2C that we compare the frequency of CD73^+^ T cells in different cohorts of HIV-infected individuals, the rest of our studies were conducted on samples from HIV-infected individuals on ART due to the availability of larger sample size.

### Cell isolation and processing

Peripheral blood mononuclear cells (PBMCs) were isolated from the blood of either HIV-infected or HCs using Ficoll-Paque gradients. Cell cultures were performed in RPMI 1640 (Sigma-Aldrich) supplemented with 10% FBS (Sigma-Aldrich) and 1% penicillin/streptomycin (Sigma-Aldrich). Our studies were mainly conducted on freshly isolated cells but occasionally cryopreserved PBMCs were used. In some studies, total T cells or CD8^+^ T cells were isolated from PBMCs according to the manufacturer’s instructions (STEMCELL Technologies), with a purity exceeding 95% (S3L Fig). In other studies, CD73^-^ and CD73^+^CD8^+^ T cells were isolated using MACS separation columns after labelling CD8^+^ T cells with the biotin-conjugated anti–CD73 mAb (AD2), followed by the anti-biotin microbeads (Miltenyi Biotec), and passed through MACS separation columns (Miltenyi Biotec) (S3M Fig) as reported elsewhere [75].

For the effector T cell isolation, CCR7 negative total T cells were isolated by negative selection. Briefly, total T cells were labeled with the FITC-conjugated anti-CCR7 and passed through MACS separation columns (Miltenyi Biotec) to isolate the negative fraction (S3N Fig). For the proliferation assay, isolated effector T cells were labeled with 1.25 μM CFSE (Thermo Fisher Scientific) as described elsewhere [34,76] before stimulation with anti-CD3/CD28 microbeads for 3–4 days. In some experiments, isolated CD8^+^ T cells were cultured with the anti-CD3 (3μg/mL) and anti-CD28 (1μg/mL) antibodies in the presence or absence of recombinant IL-2 (NIH HIV-reagents), IL-15 (BioLegend), IL-16 (R&D), TNF-α (R&D), IFN-α (Abcam), IL-10 (R&D), TGF-β (BioLegend), IFN-γ (STEMCELL Technologies) and IL-15 (BioLegend). In other experiments, CD8^+^ T cells were cultured with ATP (25 μM) (Thermo Fisher Scientific).

### Cell culture and flow cytometry

Fluorophore-labelled antibodies with specificity to human cell antigens and cytokines were purchased mainly from BD Biosciences, Thermo Fisher Scientific, BioLegend, R&D and abcam. The following Abs were used in our study: anti-CD3 (SK7), anti-CD4 (RPA-T4), anti-CD8 (RPA-T8), anti-CD39 (TU66), anti-CD107a (H4A3), anti-Perforin (dG9), anti–Granzyme B (GzmB; GB11), anti-CD45RA (HL100), anti-CD62L (DREG-56), anti-CD73 (AD2), anti-CCR7 (2-L1-A), anti-FasL (NOK-1), anti-IL-2 (MQ1-17H12), anti-TNF-α (MAB11), anti-IFN-γ (4S.B3), anti-Integrin β7 (FIB504), anti-P2X1/P2RX1 (551820) and Alexa Fluor-conjugated anti-mouse IgG secondary antibody (ab150113). For defining the gating strategy and antibody specificity, appropriate FMO (fluorescence minus one) and isotype control antibodies were used per the supplier’s recommendation. Also, BD Biosciences CompBeads were used for control compensation. Purified anti-human CD3 (UCHT1), anti-human CD28 (CD28.2), Protein Transport Inhibitor (containing brefeldin A), and Protein Transport Inhibitor (containing monensin) were purchased from the BD Biosciences. Cell stimulation mixture (PMA/ionomycin) was purchased from BioLegend. Surface and intracytoplasmic cytokine staining (ICS) was performed according to our previous reports [77–79]. For ICS, depending on the experiment, PMBCs were cultured and stimulated with a HIV-derived Gag peptide pool, PMA (2 ng/ml), or anti-CD3 (3 μg/mL) and anti-CD28 (1 μg/mL) antibodies for 5 hr in the presence of Brefeldin A (1 μg/ml). CD107a staining was performed as described elsewhere [52]. For measuring surface versus intracellular CD73 expression, we used different anti-CD73 Fluorochromes for surface and intracellular staining. Where specified, the P2X antagonist pyridoxal-phosphate-6-azophenyl-2’-4’disulfonate (PPAD) was added to the medium (20 μM and 50 μM, Sigma-Aldrich) for 48 hr.

Cells were fixed and permeabilized and acquired on an LSRFortessa-SORP or Fortessa- X20 (BD Biosciences) and analyzed using the FlowJo software (version 10).

### T cell migration assay

Cell migration was assessed using the CytoSelect migration assay kit (Cell Biolabs, Cat# CBA-104). CD8^+^ T cells were isolated followed by sorting CD73^+^ and CD73^-^ effector T cells. We cultured both effector CD8^+^CD73^+^ and CD8^+^CD73^-^ T cells in 96-well migration plate at 0.5 x 10^6^ cells/well overnight in the absence of FBS. The next day, RPMI culture media containing 10% FBS was added in the bottom chamber as the chemoattractant. The cell migration was calculated by the quantification of the fluorescence using a fluorescence plate reader according to the manufacturer’s instructions as we have reported elsewhere [80].

### Gene expression analysis

The RNA was isolated from approximately 1×10^6^ CD8^+^ T cells using the Direct-zol RNA MicroPrep kit (Zymo Research). cDNA (100 ng) samples for miRNA and mRNA expression were synthesised using the miScript II RT kit (Qiagen), as described previously (27). cDNA samples for mRNA expression using RT^2^ primers were synthesised using the RT^2^ Reverse Transcription kit (Qiagen). For quantitative real-time PCR (RT-PCR), samples were run in duplicate using Quantitect or and RT^2^ Primer Assays (mRNAs) and miScript Primer Assays (miRNAs) (Qiagen) using the CFX96 Touch Real-Time PCR Detection System (BioRad). Expression of the following mRNAs and miRNAs were analysed: *NT5E*, *TNFA*, *IFNG*, *IL2*, *and FoxP3*, *PRF*, *GZMB*, miR-30a, -30b, -30c, -30d, and -30e. Beta-2-microglobulin and RNU6 were used as a reference gene for mRNAs and miRNAs, respectively. Data analysis was carried out using the 2 ^-ddCT^ method as we reported elsewhere [81,82].

### Cell nucleofection

CD8^+^ T cells were stimulated with the anti-CD3 (3 μg/mL), anti-CD28 (1 μg/mL) and IL-2 (25 IU/mL) for 48 hours. The cell pellet (2 × 10^6^ cells) was resuspended in 100 μl of P3 Primary Cell Nucleofector Solution (Lonza). Using the stimulated human T cells program of the 4D Nucleofactor ^TM^ Core Unit (Lonza). Nucleofected cells were resuspended in 400 μl of prewarmed X-VIVO 15 medium (Lonza) in 24-well plate for 48 hours. Changes in miRNAs were confirmed by RT-PCR.

### ELISA

The concentration of CD73 in the plasma of HIV-infected and healthy controls was measured using an ELISA kit (R&D Systems).

### ATP assay

The ATP concentration was measured in the plasma using the ATPlite luminescence assay system (PerkinElmer, MA). Plasma samples (100 μL) were combined with 100 μL of ATPlite reagent and the Luminescence was measured using a BioTek Synergy H1 Multi-Mode Reader. The concentration of ATP was calculated by comparing the luminescence of the samples to a standard curve generated using ATP standards according to the manufacture’s instruction.

### Statistical analysis

We initially determined the distribution of data using the Wilks-Shapiro test and then based on the distribution of data the appropriate test was used. When data were not normally distributed the non-parametric tests such as the Mann-Whitney U-test or Kruskal–Wallis one-way analysis of variance was used. The *P*-values are shown in the graphs and measures are expressed as mean ± SEM and *P-*value < 0.05 was considered to be statistically significant.

## Supporting information

S1 Fig (**A**) The gating strategy for CD4^+^/CD8^+^ T cells. (**B**) Representative plots of CD73 expression in CD3^+^/CD3^-^ cells. (**C**) Cumulative results of the plasma CD73 levels in HCs and HIV-infected individuals. (**D**) Cumulative data for TNF-α, IFN-γ and IL-2 expression in CD8^+^CD73^-^ versus CD8^+^CD73^+^ T cells from HCs following stimulation with anti-CD3/CD28 and (**E**) the same cytokines in PBMCs of HIV-infected individuals after stimulation with PMA for 6 hr as measured by ICS. (**F**) Representative plots of TNF-α and IFN-γ expression in different subsets of CD8^+^CD73^+^ or CD8^+^CD73^-^ T cells. Each dot represents results from a human subject. Data are obtained from multiple independent experiments. Statistical analysis determined by the Mann-Whitney U-test (F-H).
(TIF)

S2 Fig (**A**) Representative flow cytometry plots, and (**B**, **C**) cumulative data of cytokine expression in isolated CD8^+^CD73^-^ versus CD8^+^CD73^+^ T cells upon stimulation with anti-CD3/CD28 antibodies for 6 hr as measured by ICS. (**D**) Cumulative data of HLA-DR and CD38 expression on CD73^-^/CD73^+^CD8^+^ T cells of HIV-infected individuals. (**E**) Representative flow plots of CD8^+^CD73^+^ T cells in CSF of a MS patient at the remission and relapse times. (**F**) Cumulative data of percentages of CD73^+^CD8^+^ T cells in CSF of four MS patients while on remission and three MS patients upon relapse. Each dot represents a human subject either HIV-infected individual or MS patient. Data are obtained from multiple independent experiments. Statistical analysis determined by the Mann-Whitney U-test (B-D, F). ns: no significant. Each dot represents data from a study subject.
(TIF)

S3 Fig (**A**) Cumulative data showing fold regulation of CD73 gene in CD8^+^ T cells in either unstimulated (Unstim), stimulated (Stim) or stimulated plus IL-2 (50 IU/ml). (**B**) Cumulative data of fold regulation of CD73 gene in CD8^+^ T cells in either unstimulated, stimulated or stimulated plus IL-15 (100 ng/ml). (**C**) Cumulative data of fold regulation of CD73 gene in CD8^+^ T cells in either unstimulated, stimulated or stimulated plus IL-16 (1 μg/ml). (**D**) Cumulative data of fold regulation of CD73 gene in CD8^+^ T cells in either unstimulated, stimulated or stimulated plus TNF-α (50 ng/ml). (**E**) Cumulative data showing fold regulation of CD73 gene in CD8^+^ T cells in either unstimulated, stimulated or stimulated plus IFN-α (100 ng/ml). (**F**) Cumulative data showing fold regulation of CD73 gene in CD8^+^ T cells in either unstimulated, stimulated or stimulated plus IL-10 (100 ng/ml). (**G**) Cumulative data of fold regulation of CD73 gene in CD8^+^ T cells in either unstimulated, stimulated or stimulated plus TGF-beta (20 ng/ml). (**H-K**) Fold regulation of miRNA30b-30e in isolated CD8+ T cells after their treatment with their corresponding miRNA-inhibitors quantified by qPCR. Data are obtained from multiple independent experiments. Statistical analysis determined by the Mann-Whitney U-test (A-E, H-K) and Kruskal–Wallis test (F and G). **(L**) The representative histogram plot showing the purity of total CD8^+^, (**M**) CD73^-^, CD73^+^, and **(N**) effector CD8^+^ T cells.
(TIFF)

S1 Table Showing patients clinical information (CD4 count and viral load) and disease status (e.g. Long-term non-progressors (LTNPs), on antiretroviral therapy (ART) and those who have never been on ART (ART-naïve) or newly diagnosed. Male (M) and female (F).
(DOCX)
