# Supplementary figures and images for: Elevated ATP via enhanced miRNA-30b, 30c, and 30e downregulates the expression of CD73 in CD8+ T cells of HIV-infected individuals
Source: PLoS Pathog. 2022 Mar 24;18(3):e1010378. doi: 10.1371/journal.ppat.1010378 (PMC8947394; doi:10.1371/journal.ppat.1010378)

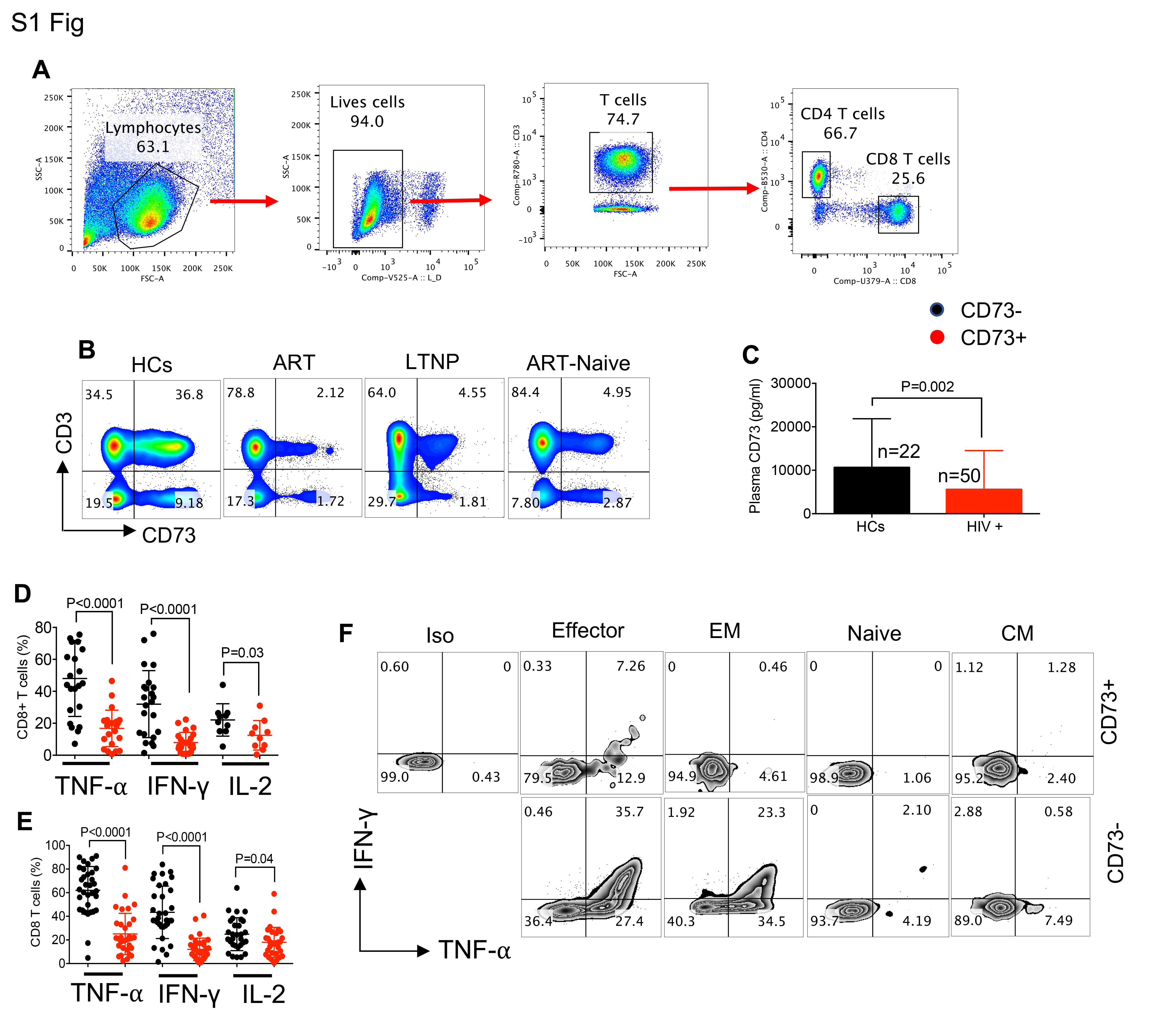

Supplement: S1 Fig — (A) The gating strategy for CD4+/CD8+ T cells. (B) Representative plots of CD73 expression in CD3+/CD3- cells. (C) Cumulative results of the plasma CD73 levels in HCs and HIV-infected individuals. (D) Cumulative data for TNF-α, IFN-γ and IL-2 expression in CD8+CD73- versus CD8+CD73+ T cells from HCs following stimulation with anti-CD3/CD28 and (E) the same cytokines in PBMCs of HIV-infected individuals after stimulation with PMA for 6 hr as measured by ICS. (F) Representative plots of TNF-α and IFN-γ expression in different subsets of CD8+CD73+ or CD8+CD73- T cells. Each dot represents results from a human subject. Data are obtained from multiple independent experiments. Statistical analysis determined by the Mann-Whitney U-test (F-H). (TIF) [file ppat.1010378.s001.tif]

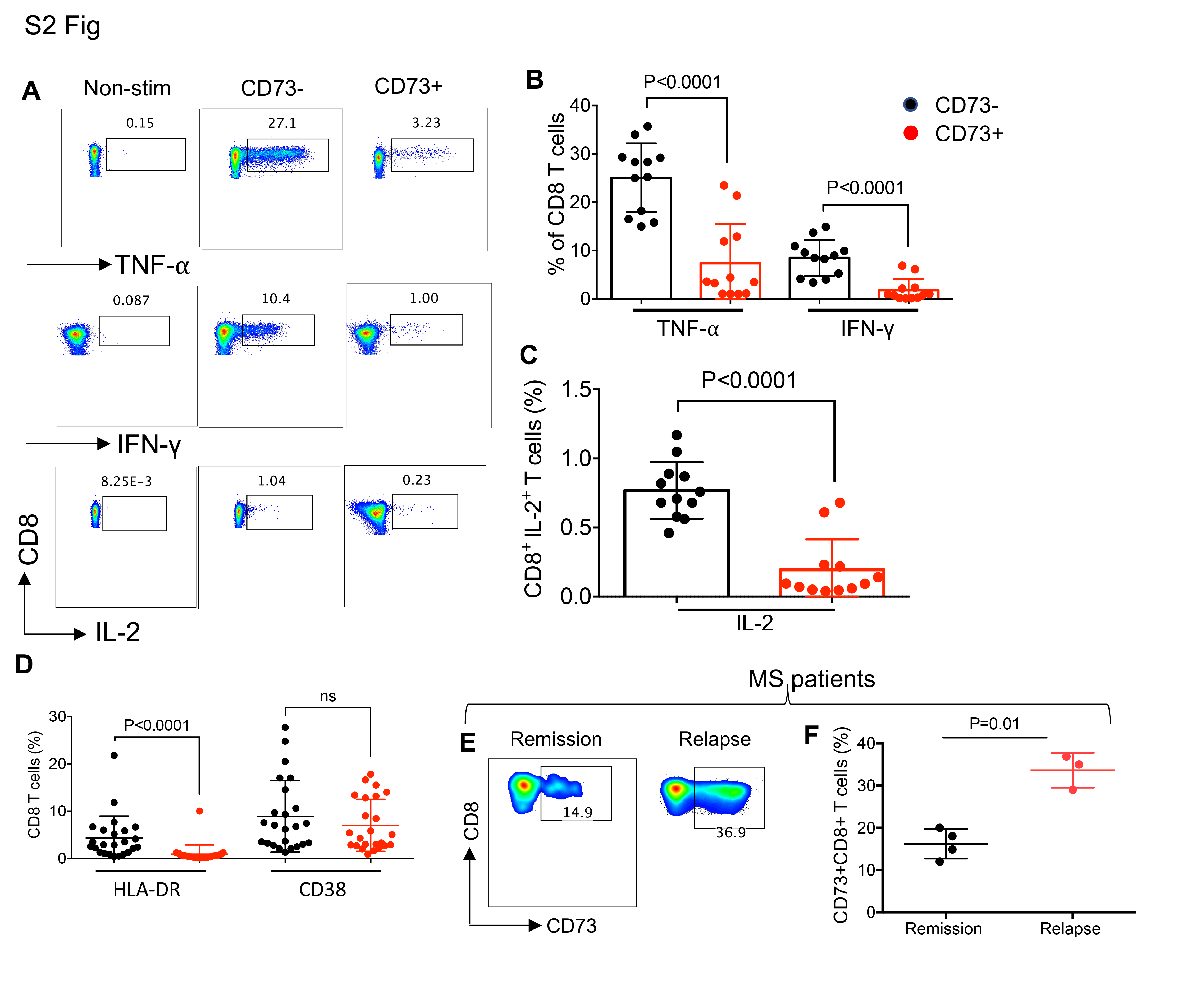

Supplement: S2 Fig — (A) Representative flow cytometry plots, and (B, C) cumulative data of cytokine expression in isolated CD8+CD73- versus CD8+CD73+ T cells upon stimulation with anti-CD3/CD28 antibodies for 6 hr as measured by ICS. (D) Cumulative data of HLA-DR and CD38 expression on CD73-/CD73+CD8+ T cells of HIV-infected individuals. (E) Representative flow plots of CD8+CD73+ T cells in CSF of a MS patient at the remission and relapse times. (F) Cumulative data of percentages of CD73+CD8+ T cells in CSF of four MS patients while on remission and three MS patients upon relapse. Each dot represents a human subject either HIV-infected individual or MS patient. Data are obtained from multiple independent experiments. Statistical analysis determined by the Mann-Whitney U-test (B-D, F). ns: no significant. Each dot represents data from a study subject. (TIF) [file ppat.1010378.s002.tif]

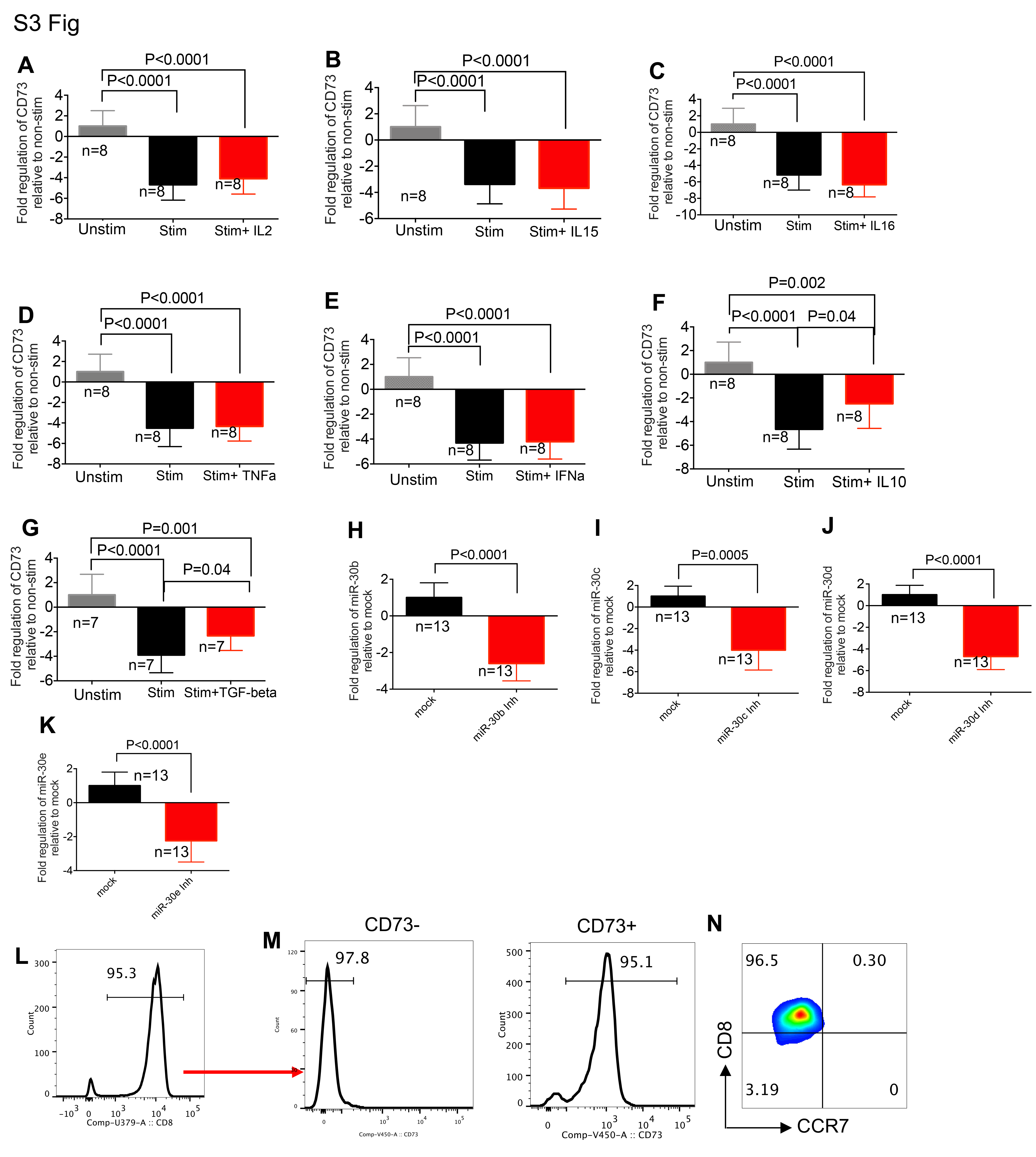

Supplement: S3 Fig — (A) Cumulative data showing fold regulation of CD73 gene in CD8+ T cells in either unstimulated (Unstim), stimulated (Stim) or stimulated plus IL-2 (50 IU/ml). (B) Cumulative data of fold regulation of CD73 gene in CD8+ T cells in either unstimulated, stimulated or stimulated plus IL-15 (100 ng/ml). (C) Cumulative data of fold regulation of CD73 gene in CD8+ T cells in either unstimulated, stimulated or stimulated plus IL-16 (1 μg/ml). (D) Cumulative data of fold regulation of CD73 gene in CD8+ T cells in either unstimulated, stimulated or stimulated plus TNF-α (50 ng/ml). (E) Cumulative data showing fold regulation of CD73 gene in CD8+ T cells in either unstimulated, stimulated or stimulated plus IFN-α (100 ng/ml). (F) Cumulative data showing fold regulation of CD73 gene in CD8+ T cells in either unstimulated, stimulated or stimulated plus IL-10 (100 ng/ml). (G) Cumulative data of fold regulation of CD73 gene in CD8+ T cells in either unstimulated, stimulated or stimulated plus TGF-beta (20 ng/ml). (H-K) Fold regulation of miRNA30b-30e in isolated CD8+ T cells after their treatment with their corresponding miRNA-inhibitors quantified by qPCR. Data are obtained from multiple independent experiments. Statistical analysis determined by the Mann-Whitney U-test (A-E, H-K) and Kruskal–Wallis test (F and G). (L) The representative histogram plot showing the purity of total CD8+, (M) CD73-, CD73+, and (N) effector CD8+ T cells. (TIFF) [file ppat.1010378.s003.tiff]
