# Supplementary material for: Elevated ATP via enhanced miRNA-30b, 30c, and 30e downregulates the expression of CD73 in CD8+ T cells of HIV-infected individuals
Source: PLoS Pathog. 2022 Mar 24;18(3):e1010378. doi: 10.1371/journal.ppat.1010378 (PMC8947394; doi:10.1371/journal.ppat.1010378)
Supplement: S1 Table — Male (M) and female (F). (DOCX) [file ppat.1010378.s004.docx]

**Supplementary Table 1** Participants demographic and clinical data.

| PTID | Sex | Plasma viral load (copies/mL-^1^) | CD4 count  (mL^-1^) | On ART |
| --- | --- | --- | --- | --- |
| LTNP-1 | M | 792 | 570 | no |
| LTNP-2 | M | <50 | 522 | no |
| LTNP-3 | M | <50 | 473 | no |
| LTNP-4 | F | 800 | 421 | no |
| LTNP-5 | M | <50 | 796 | no |
| LTNP-6 | M | <50 | 832 | no |
| LTNP-7 | M | 125 | 783 | no |
| LTNP-8 | M | <50 | 759 | no |
| LTNP-9 | F | <50 | 773 | no |
| LTNP-10 | M | 65 | 426 | no |
| LTNP-11 | F | <50 | 643 | no |
| LTNP-12 | F | 1073 | 574 | no |
| LTNP-13 | M | 140 | 555 | no |
| ART-1 | M | <30 | 720 | yes |
| ART-2 | M | <30 | 570 | yes |
| ART-3 | M | <30 | 1080 | yes |
| ART-4 | M | <30 | 450 | yes |
| ART-5 | M | 1399 | 800 | yes |
| ART-6 | M | <30 | 720 | yes |
| ART-7 | M | <30 | 830 | yes |
| ART-8 | M | <30 | 460 | yes |
| ART-9 | M | <30 | 470 | yes |
| ART-10 | F | <30 | 410 | yes |
| ART-11 | M | <30 | 350 | yes |
| ART-12 | M | <30 | 850 | yes |
| ART-13 | M | <30 | 740 | yes |
| ART-14 | M | <30 | 680 | yes |
| ART-15 | M | <30 | 470 | yes |
| ART-16 | M | <30 | 550 | yes |
| ART-17 | M | 208 | 410 | yes |
| ART-18 | M | <30 | 420 | yes |
| ART-19 | M | <30 | 580 | yes |
| ART-20 | F | <30 | 590 | yes |
| ART-21 | M | <30 | 680 | yes |
| ART-22 | M | <30 | 570 | yes |
| ART-23 | M | <30 | 420 | yes |
| ART-24 | M | <30 | 210 | yes |
| ART-25 | M | <30 | 500 | yes |
| ART-26 | M | <30 | 640 | yes |
| ART-27 | M | <30 | 260 | yes |
| ART-28 | F | <30 | 700 | yes |
| ART-29 | F | <30 | 720 | yes |
| ART-30 | M | <30 | 910 | yes |
| ART-31 | F | 168 | 290 | yes |
| ART-32 | F | <30 | 180 | yes |
| ART-33 | M | <30 | 680 | yes |
| ART-34 | M | <30 | 510 | yes |
| ART-35 | M | <30 | 1210 | yes |
| ART-36 | F | <30 | 550 | yes |
| ART-37 | F | 60 | 910 | yes |
| ART-38 | F | <30 | 190 | yes |
| ART-39 | M | <30 | 340 | yes |
| ART-40 | F | 160 | 60 | yes |
| ART-41 | M | <30 | 390 | yes |
| ART-42 | M | <30 | 250 | yes |
| ART-43 | M | <30 | 520 | yes |
| ART-44 | M | <30 | 610 | yes |
| ART-45 | M | <30 | 360 | yes |
| ART-46 | M | <30 | 290 | yes |
| ART-47 | M | <30 | 250 | yes |
| ART-48 | M | <30 | 260 | yes |
| AET-49 | M | <30 | 342 | yes |
| ART-50 | F | <30 | 457 | yes |
| ART-51 | M | <30 | 632 | yes |
| ART-52 | M | 540 | 578 | yes |
| ART-53 | M | 990 | 550 | yes |
| ART-54 | M | <30 | 602 | yes |
| ART-55 | M | <30 | 575 | yes |
| ART-56 | F | 800 | 451 | yes |
| ART-57 | M | <30 | 790 | yes |
| ART-58 | M | <30 | 850 | yes |
| ART-59 | M | 125 | 783 | yes |
| ART-60 | M | <30 | 765 | yes |
| ART-61 | M | <30 | 562 | yes |
| ART-62 | F | <30 | 690 | yes |
| ART-63 | F | <30 | 720 | yes |
| ART-naïve-1 | F | 88800 | 307 | No |
| ART-naïve-2 | F | 20000 | 333 | No |
| ART-naïve-3 | M | 40370 | 463 | No |
| ART-naïve-4 | F | 368000 | 305 | No |
| ART-naïve-5 | M | 15200 | 447 | No |
| ART-naïve-6 | M | 172200 | 479 | No |
| ART-naïve-7 | M | 32300 | 798 | No |
| ART-naïve-8 | M | 73300 | 156 | No |
| ART-naïve-9 | M | 17200 | 382 | No |
| ART-naïve-10 | F | 18800 | 390 | No |
| ART-naïve-11 | M | 37400 | 335 | No |
| ART-naïve-12 | M | 13700 | 414 | No |
| ART-naïve-13 | M | 25300 | 259 | No |
| ART-naïve-14 | F | 383000 | 551 | No |
| ART-naïve-15 | M | 153000 | 329 | No |
| ART-naïve-16 | M | 385000 | 239 | No |
| ART-naïve-17 | M | 93300 | 396 | No |
| ART-naïve-18 | M | 153000 | 321 | No |
| ART-naïve-19 | M | 121000 | 408 | No |
| ART-naïve-20 | F | 19500 | 637 | No |
| ART-naïve-21 | M | 40100 | 748 | No |
| ART-naïve-22 | M | 476000 | 556 | No |
| ART-naïve-23 | M | 225000 | 621 | No |
| ART-naïve-24 | M | 71500 | 547 | No |
| ART-naïve-25 | M | 67200 | 448 | No |
| ART-naïve-26 | F | 24100 | 787 | No |
